# THERMORESPONSIVE, REDOX-POLYMERIZED CELLULOSIC HYDROGELS UNDERGO *IN SITU* GELATION AND RESTORE INTERVERTEBRAL DISC BIOMECHANICS POST DISCECTOMY

**DOI:** 10.22203/eCM.v035a21

**Published:** 2018-05-30

**Authors:** D.M. Varma, H.A. Lin, R.G. Long, G.T. Gold, A.C. Hecht, J.C. Iatridis, S.B. Nicoll

**Affiliations:** 1 Department of Biomedical Engineering, The City College of New York, New York, NY, USA; 2 Leni and Peter W. May Department of Orthopedics, Icahn School of Medicine at Mount Sinai, New York, NY, USA

**Keywords:** Intervertebral disc, nucleus pulposus, cellulosic hydrogel, injectable, discectomy

## Abstract

Back and neck pain are commonly associated with intervertebral disc (IVD) degeneration. Structural augmentation of diseased nucleus pulposus (NP) tissue with biomaterials could restore degeneration-related IVD height loss and degraded biomechanical behaviors; however, effective NP replacement biomaterials are not commercially available. This study developed a novel, crosslinked, dual-polymer network (DPN) hydrogel comprised of methacrylated carboxymethylcellulose (CMC) and methylcellulose (MC), and used *in vitro, in situ* and *in vivo* testing to assess its efficacy as an injectable, *in situ* gelling, biocompatible material that matches native NP properties and restores IVD biomechanical behaviors. Thermogelling MC was required to enable consistent and timely gelation of CMC *in situ* within whole IVDs. The CMC-MC hydrogel was tuned to match compressive and swelling NP tissue properties. When injected into whole IVDs after discectomy injury, CMC-MC restored IVD height and compressive biomechanical behaviors, including range of motion and neutral zone stiffness, to intact levels. Subcutaneous implantation of the hydrogels in rats further demonstrated good biocompatibility of CMC-MC with a relatively thin fibrous capsule, similar to comparable biomaterials. In conclusion, CMC-MC is an injectable, tunable and biocompatible hydrogel with strong potential to be used as an NP replacement biomaterial since it can gel *in situ*, match NP properties, and restore IVD height and biomechanical function. Future investigations will evaluate herniation risk under severe loading conditions and assess long-term *in vivo* performance.

## Introduction

Intervertebral disc (IVD) degeneration is strongly associated with back pain, affecting 15–30 % of the US population at estimated annual costs of $194 billion (Becker *et al.*, 2010; Jacobs, 2008). The IVD consists of the centrally located nucleus pulposus (NP) and peripherally located annulus fibrosus (AF). The NP is a highly hydrated tissue that functions to bear compressive loads and maintain the IVD height by means of swelling pressures. The AF resists spinal motions and NP swelling by bearing tensile stresses through its fibrous and laminated structure. With IVD degeneration there is a loss of constituent matrix molecules, such as proteoglycans and collagen, leading to non-uniform load distributions in the IVD that manifest as cracks and fissures in the AF and can result in herniation (Adams *et al.*, 1996). IVD herniation is a common condition that is characterized by extrusion of the NP through the AF and is highly associated with clinically painful conditions (Endean *et al.*, 2011; Weber, 1994). Discectomy is a procedure widely performed to treat IVD herniations and involves the removal of the extruded NP tissue to prevent its contact with surrounding nerves. It is clinically favorable to non-operative controls in providing pain relief but does not restore IVD biomechanical behaviors that are altered by the loss in IVD height and intradiscal pressure, which result from NP herniation and AF damage. There is also a recurrence rate of 5–15 % (Kim *et al.*, 2009; McGirt *et al.*, 2009; Shin, 2014). Advancement of IVD degeneration following damage, herniation and/or discectomy may require patients to undergo spinal fusion, which also has deleterious biomechanical consequences on motion segments adjacent to the treated segments (Kim *et al.*, 2009). Furthermore, recent explant studies indicate that degenerated IVDs that undergo discectomy are more susceptible to hypermobility in the motion segment, in turn increasing spinal instability (Showalter *et al.*, 2014). All of these conditions highlight a need for biomaterial-based strategies that are capable of repairing and restoring function of injured and degenerated IVDs.

Replacing degraded or herniated NP tissue with a biomaterial could limit progression of degeneration and reduce painful conditions. NP replacement strategies have evolved with early generations employing preformed devices that required invasive procedures for implantation. Many devices failed clinically due to device migration and extrusion from the herniation site (Lewis, 2012; Di Martino *et al.*, 2005). AF sealants and closure devices can address device migration concerns (Guterl *et al.*, 2013; Parker *et al.*, 2016). However, AF repair strategies alone may not fully restore IVD biomechanics. Recently developed NP replacements include hydrogels, because of their high water-content, tunable material properties, and ability to conform to the intradiscal space (Balkovec *et al.*, 2013; Gan *et al.*, 2017; Pérez-San Vicente *et al.*, 2017; Showalter *et al.*, 2015).

Design objectives for an ideal hydrogel-based, NP replacement biomaterial include: injectability with rapid *in situ* gelation, appropriate for a minimally invasive clinical setting; stable formation of gels that mimic healthy NP material properties, restoring IVD height and multiple biomechanical behaviors of injured IVDs; and biocompatibility (Iatridis *et al.*, 2013). Although injectable hydrogels have been developed, few studies have assessed the ability of such gels to restore disc mechanical function. Cannella *et al.* demonstrate restoration of select biomechanical properties with physically crosslinked polyvinyl alcohol (PVA)-polyvinylpyrrolidone hydrogels (Cannella *et al.*, 2014). Generally, PVA hydrogels are pre-crosslinked by means of physical crosslinks formed using repetitive freeze-thaw cycles (Cannella *et al.*, 2014; Joshi *et al.*, 2006) or chemically, using crosslinkers such as diglycidyl ether (Binetti *et al.*, 2014). They have limited capability to conform to the disc space post injury or nucleotomy, in comparison to *in situ* gelling materials. As a result, these hydrogels may be more susceptible to herniation. An *in situ* gelling tripolymeric hydrogel developed by Smith *et al.* exhibits robust mechanical properties, and restores compressive biomechanics in a fatigue model and a preclinical model (Gullbrand *et al.*, 2017; Malhotra *et al.*, 2012; Showalter *et al.*, 2015; Smith *et al.*, 2014). However, this Schiff base-dependent hydrogel requires >10 h of crosslinking time to achieve peak biomechanical properties, which may limit its clinical translation, given that rapid gelation is a key requirement.

Carboxymethyl cellulose (CMC)-based hydrogels can meet many of the design criteria for NP replacements. CMC is a water-soluble derivative of cellulose with an extensive safety and biocompatibility profile, as it has been FDA-approved for use in the pharmaceutical and food industries (Heinze and Koschella, 2005). CMC mimics the NP, with an exaggerated water absorbing capacity at physiological pH, due to the carboxyl groups on the polymer backbone. Unlike other commonly used biopolymers (such as collagen and hyaluronic acid), CMC does not undergo enzymatic degradation in humans, providing enhanced stability. Moreover, modification of the hydroxyl groups along the CMC backbone with methacrylate moieties allows covalent coupling of the polymer chains in the presence of radical initiators, forming stable crosslinked hydrogels (Reza and Nicoll, 2010). *In vitro* characterization of photocrosslinked methacrylated CMC demonstrates that CMC hydrogels have functional properties comparable to native NP and can support stem cell differentiation towards an NP phenotype (Gupta *et al.*, 2011; Gupta and Nicoll, 2013; Gupta and Nicoll, 2014; Lin *et al.*, 2016). However, UV-based crosslinking methods have limited clinical applicability for IVD repair due to poor deep tissue penetration and potential harm to the surrounding tissue.

**Fig. 1:**
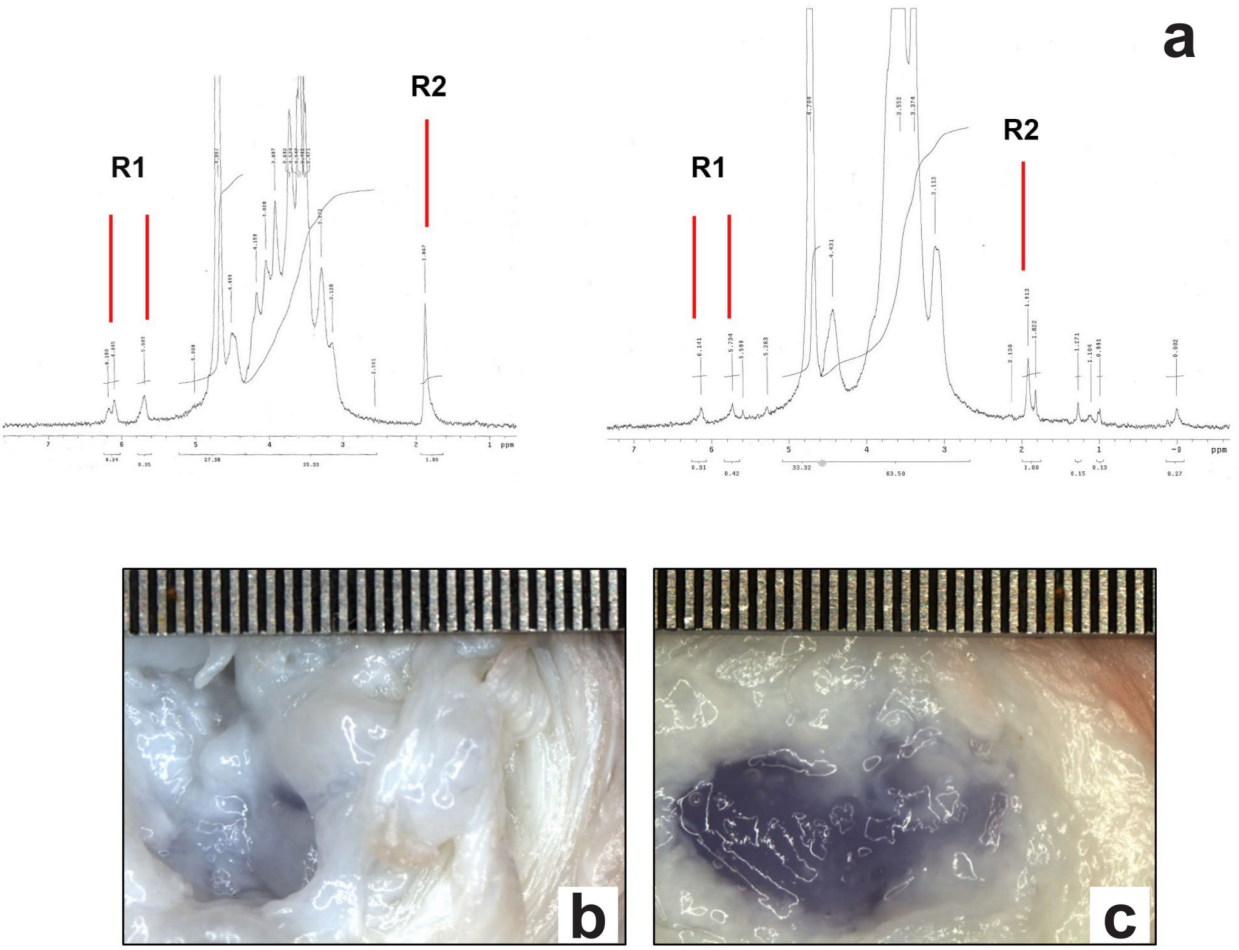
*In situ* gelation within nucleotomized IVDs occurred with redox-initiated CMC-MC crosslinked DPN but not with CMC alone. (**a**) Representative ^1^H-NMR spectra of CMC (left) and MC (right) modified with methacrylate groups on the polymer backbone. R1 and R2 indicate the methylene and methyl protons on the methacrylate group, respectively. (**b**) No *in situ* gelation was observed using redox-initiated CMC, as exemplified by a 3 % (w/v) 90 kDa CMC solution alone with 20 mm APS/TEMED initiators, where a slight blue-coloring occurred in the native NP region of the IVD which had been disrupted from discectomy. (**c**) *In situ* gelation always occurred with CMC-MC DPN, as shown by a 3 % (w/v) 90 kDa CMC- 3 % (w/v) 15 kDa : 41 kDa (1 : 1) MC solution with 20 mm APS/TEMED initiators, wherein a blue gel had filled the NP region after discectomy. Trypan blue dye was used for visualization since CMC and MC are clear. Scale in mm.

The development and characterization of an injectable CMC-based hydrogel for NP repair is an important step required to advance towards clinical translation. A fully injectable CMC hydrogel was previously developed by covalently crosslinking with water-soluble redox initiators, ammonium persulfate (APS) and tetramethylethylene diamine (TEMED). This redox-initiated CMC hydrogel matches NP properties and forms stable hydrogels *in vitro* (Varma, 2016). However, redox-polymerized CMC failed to gel when injected into large IVDs *in situ* post discectomy, due to extravasation of the hydrogel solution from the IVD upon injection. To overcome this challenge to clinical translation, a novel dual-polymer network (DPN) consisting of modified CMC and methacrylated methylcellulose (MC) was developed. The inherent thermogelation property of MC at > 32 °C increased the viscosity of the redox-initiated DPN solution at body temperature, enabling consistent and reliable gelation *in situ* in the microenvironment of large IVDs (Fig. 1). As such, the objectives of this study were to: 1) develop a novel injectable, thermogelling, redox-polymerized CMC-MC hydrogel with material properties akin to the native NP; 2) determine if the CMC-MC hydrogel could restore native biomechanics of the bovine IVD post discectomy; and 3) assess CMC-MC hydrogel cytocompatibility using 3D cell culture and biocompatibility in a rat subcutaneous pouch model.

## Materials and Methods

### Polymer synthesis

High and low molecular weights of CMC (Sigma-Aldrich) at 250 kDa and 90 kDa, were used in combination with an MC (Sigma-Aldrich) polymer blend of two molecular weights, 15 kDa and 41 kDa, in a 1 : 1 ratio for this study. The low-high molecular weight blend of MC was selected to provide adequate control over solution viscosity and handling. Methacrylation of CMC and MC polymers was performed by the esterification of hydroxyl groups with methacrylic anhydride (Sigma-Aldrich) at a pH of 8.5, as described by Reza and Nicoll (2010). The CMC and MC reaction products were purified by dialysis and the degree of methacrylation was quantified using ^1^H-NMR (300 MHz, Varian Mercury 300) following acid hydrolysis of both modified polymers (Gold *et al.*, 2014; Varma *et al.*, 2014). Briefly, 20 mg of the modified polymer dissolved in 20 mL of deionized water was hydrolyzed at a pH of 2 for 2–3 h at 80 °C. After cooling, the pH of the solutions was adjusted to 7 and the hydrolyzed polymer was extracted after lyophilization. The dried product was dissolved in 1 mL of deuterium dioxide solvent and analyzed for methacrylate peaks using ^1^H-NMR. Molar percentage of methacrylation was determined by the relative integrations of the methacrylate proton peaks (methylene peak, δ = 6.2 and 5.8 ppm; methyl peak, δ = 2.0 ppm) with respect to the carbohydrate backbone.

### Part I: *In situ* gelation and hydrogel characterization

Characterization of the CMC-MC hydrogel occurred following *in situ* gelation within IVD motion segments to be consistent with the intended minimally invasive clinical application of the biomaterial. Bovine caudal IVDs were used since they are readily available, commonly accepted in the literature, contain a fibrous NP and exhibit several properties similar to human IVDs (Alini *et al.*, 2008). Discectomy was performed on healthy bovine IVDs to approximate current clinical practice as well as to produce a void into which the hydrogel could be injected. The resulting biomechanical changes have been found to mimic IVDs undergoing degeneration (Showalter *et al.*, 2014). CMC-MC DPNs were then injected *in situ* into the IVDs, before being isolated and prepared to uniform geometry for *in vitro* hydrogel characterization tests.

#### Bovine explant sample preparation and discectomy injury

Bone-disc-bone motion segments (cc2–3 and cc3–4) were harvested from healthy, skeletally mature bovine tails obtained from a local abattoir. The segments were prepared by sawing parallel cuts through the vertebral bodies (Likhitpanichkul *et al.*, 2014). A cruciate incision was made posterolaterally with a #15 blade through the AF. The NP was dislodged with a curette and approximately 0.15–0.20 g (≈ 80 %) of NP tissue was removed with a pituitary rongeur *via* the 2 mm incision (Malhotra *et al.*, 2012).

#### In situ hydrogel fabrication

CMC-MC formulations of varying macromer concentrations, molecular weights and redox initiator concentrations, as listed in Table 1, were screened for their ability to gel *in situ* within injured bovine motion segments post injection through a 20G needle coupled with a dual-barrel syringe and mixing tip. A custom-casting device was used to fabricate hydrogels *in vitro* for comparison to *in situ* cured gels.

Solutions of methacrylated CMC and MC in Dulbecco’s Phosphate Buffered Saline (DPBS) (Thermo Fisher Scientific, USA) were combined and prepared in dual-barrel syringes (Pearson Dental Supply Co., Sylmar, CA, USA). The redox initiators, APS and TEMED, were added to separate barrels of the syringe such that they mixed in the mixing tip upon injection. Trypan blue was added to detect the presence of the hydrogel in the motion segment post gelation. The uncrosslinked mixture was warmed to 37 °C in a water bath and injected with a 20 G needle (Becton, Dickinson & Co., Franklin Lakes, NJ, USA) into the NP void of the injured motion segments *via* the injury site. The implanted motion segments were incubated in a water bath at 37 °C for 30 min.

#### Hydrogel isolation and material characterization

Following *in situ* gelation, motion segments were dissected along the endplate and the hydrogel was carefully extracted and assessed for the material properties. Upon extracting the hydrogel from the motion segment, the sample was carefully separated from the NP and the outer portion of the hydrogel was excised to exclude any of the adhering NP tissue. Cylindrical hydrogel specimens (3 mm diameter, ≈ 2 mm thickness) were cored from CMC-MC hydrogels using a 3-mm biopsy punch and a custom-made cutting guide made with blades 2 mm apart.

#### Mechanical testing

Hydrogels (*n* = 7; 1 gel each from 7 independent *in situ* gelation experiments in bovine motion segments) underwent mechanical loading in unconfined compression in a DPBS bath using an established protocol (Reza and Nicoll, 2010), consisting of a creep test (1 g tare load at 10 µm/s) followed by a multi-ramp stress relaxation (three 5 % strain ramps with relaxation intervals of 2,000 s). The average equilibrium Young’s modulus (*E_y_*) was determined as the slope of the equilibrium stress *versus* strain curves at 5 %, 10 % and 15 % strain. The equilibrium stress (σ_*eq*_) and peak stress (σ_*pk*_) were calculated at the 15 % strain ramp and were used to calculate the % relaxation.

$$\%\text{relaxation}=1-\frac{\sigma_{\text{eq}}}{\sigma_{\text{pk}}}$$

The same protocol was used to measure the *E_y_* of CMC_90_MC hydrogels formed within a casting device.

#### Swelling ratio

The wet weight (*W_s_*) of the cored hydrogels was measured after an overnight incubation in DPBS at 37 °C. The gels were lyophilized to obtain the dry weights (*W_d_*) (*n* = 7). The swelling ratio, (*Q_w_*), was obtained as follows (Reza and Nicoll, 2010):

$$Q_{w}=\frac{W_{s}}{W_{d}}$$

#### Rheology

Rheological analysis was performed on the base polymers, methacrylated CMC_90_ and CMC_250_, at 37 °C to determine the impact of the polymer molecular weight on the injectability and mixing of their corresponding DPN with methacrylated MC. An AR2000ex (TA Instruments) rheometer equipped with a cone and plate geometry (2°, 20 mm) was used to record measurements. Optimal test parameters (1 % strain at 1 Hz frequency) were selected by means of strain and frequency sweep measurements on the base polymer solution (3 % (w/v)) in DPBS. The test parameters were obtained from the linear viscoelastic region where G’ and G” are independent of frequency and strain (Winter and Chambon, 1986). The complex viscosity values were obtained from a time sweep conducted over 300 s.

A similar protocol was used to record the gelation kinetics and complex viscosity of the CMC-MC DPN, where gelation completion time was defined as the first time four consecutive points exhibited less than a 2 % change in G’.

#### Statistical analysis

A one-way ANOVA with a Tukey’s *post-hoc* test was used to determine the effect of CMC molecular weight on mechanical properties and *Q_w_* of the CMC-MC hydrogels and the viscosity of the base polymers (*p* < 0.05). Data represent the mean ± standard deviation (SD).

### Part II: Biomechanical restoration post discectomy

The CMC_90_MC formulation together with APS and TEMED at 20 mm had the most favorable characteristics for an NP replacement, and thus, was selected for further biomechanical evaluation.

#### Study design

**Fig. 2:**
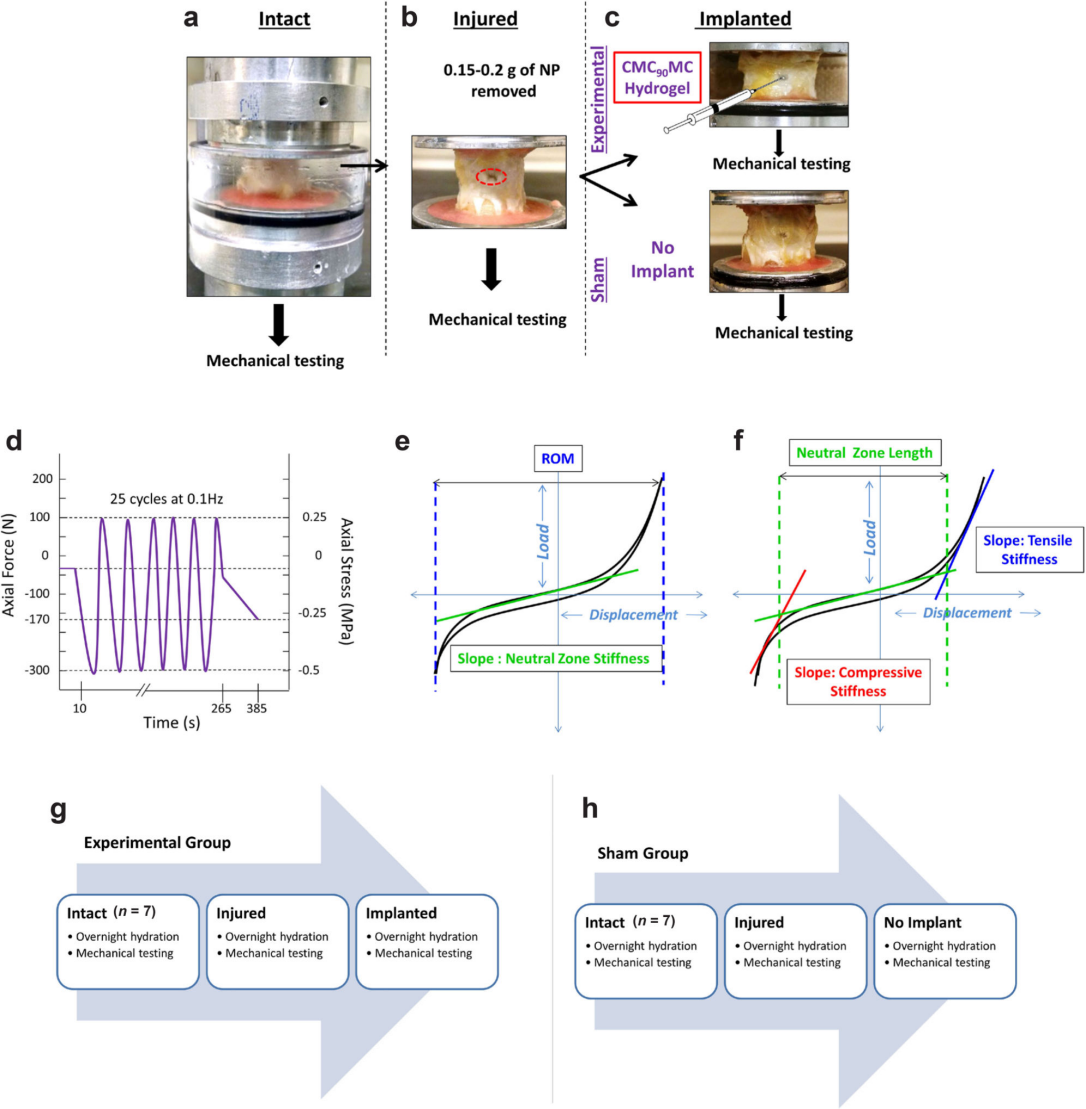
Study design and biomechanical testing protocol for bovine motion segments. The motion segments were tested using a repeated measures design including three conditions. (**a**) Intact (**b**) Injured (post discectomy) (**c**) Implanted (CMC_90_MC hydrogel implant was injected in the Experimental group while the Sham group received no implant). Representative schematics describing the (**d**) mechanical testing protocol used to test the explants at Intact, Injured and Implanted conditions. (**e**,**f**) Biomechanical parameters measured post mechanical testing. (**g** and **h**) Study regimen used for the Experimental and Sham groups over a period of 3 d.

Fourteen motion segments were randomly divided into two groups: explants receiving the CMC_90_MC hydrogel implant following injury were labeled ‘Experimental’ and designated as ‘Implanted’ in the reported results, and the control samples receiving no hydrogel implant after injury were referred to as ‘Sham’ and termed ‘No Implant’. Each motion segment underwent a mechanical loading regimen under three conditions, Intact, Injured (discectomy, as described in Part I) and Implanted/No Implant, with overnight incubation in DPBS and protease inhibitors at 4 °C. This repeated measures design (Fig. 2**a**,**b**,**c**) was used to eliminate variations between animals and IVD levels (Malhotra *et al.*, 2012). The CMC_90_MC hydrogel solution (500–750 µL) was injected into the void space of the injured IVDs in the Experimental group *via* a 20 G needle, and the motion segments (Experimental and Sham groups) were incubated at 37 °C for 30 min. The solution was injected until it exuded out of the injury site to ensure filling of the NP with the hydrogel. A radiopaque dye (Isovue^®^, Bracco, Monroe Township, NJ, USA) was mixed with the hydrogel solutions to visualize implant location post gelation.

#### Specimen preparation and mechanical testing

Seven bovine tails were used for the study with two motion segments per tail (cc2–3, cc3–4) assigned to the Experimental or Sham group. All of the musculature and soft tissue were removed, along with the facet and transverse processes, to exclude their contribution to IVD mechanics. The initial and post-test height, and average diameters of the motion segments were determined by x-ray imaging and caliper measurements, respectively, after which motion segments were potted in poly(methyl methacrylate) (Likhitpanichkul *et al.*, 2014).

Mechanical testing was performed at room temperature on an MTS servohydraulic system (Bionix 858, MTS, Eden Prairie, MN, USA), equipped with a DPBS bath. Samples were tested under load control and underwent an initial 10 s 30 N preload followed by 25 sinusoidal cycles at 0.1 Hz between-−0.5 MPa compression and 0.25 MPa tension followed by a slow ramp compression from 0 to 170 N at 1 N/s (Johannessen *et al.*, 2004; Malhotra *et al.*, 2012) (Fig. 2**d**). The peak compressive stress of 0.5 MPa was chosen based on the physiological spinal load measured in the human lumbar spine in an unsupported standing position (Wilke *et al.*, 1999), while the peak tensile stress of 0.25 MPa was sufficient to measure the NZ and tensile properties of the IVD (Likhitpanichkul *et al.*, 2014). Slow ramp compression was incorporated into the testing protocol to measure the isolated mechanical loading response of the NP, as it typically bears low loads in the IVD at low frequencies (1 N/s) (Johannessen *et al.*, 2006).

#### Biomechanical analyses

The 25^th^ cycle of the tension-compression loading regimen was used for data analysis to ensure dynamic equilibrium was attained (Johannessen *et al.*, 2006). The axial range of motion (ROM) was calculated as the total peak-to-peak displacement. A custom MATLAB (Mathworks, Natick, MA) program was used to fit the load-displacement data to a sixth order polynomial to measure the NZ parameters. The point of minimum slope on this fit was located and the NZ stiffness was calculated as the slope of the loading data at this point (Showalter *et al.*, 2014). The compressive and tensile stiffness were calculated from a linear regression of the load-displacement curve between 60–100 % of the loading curve maximum load and 80–100 % of the unloading curve maximum load, respectively (Malhotra *et al.*, 2012). The NZ length was measured as the displacement between the intersections of the compressive and tensile fits to the NZ fit. Linear regression of the slow ramp load-displacement curve was used to obtain the slow ramp compressive stiffness (Johannessen *et al.*, 2004; Johannessen *et al.*, 2006) (Fig. 2**e**,**f**). It is noteworthy that the mechanical parameters calculated from tests at 0.1 Hz have some viscoelastic contributions and should be considered ‘dynamic’ and not ‘equilibrium’ moduli parameters.

The IVD height was calculated by using a cubic spline function in a custom MATLAB code to measure the average distance between the top and bottom traced endplates on the X-Ray radiographs (Likhitpanichkul *et al.*, 2014).

#### Histology

After the third round of mechanical testing, the samples from the Experimental and Sham groups were fixed in a buffered zinc formalin solution (Z-Fix, Anatech, Battle Creek, MI, USA). The IVDs were sawed off of the vertebral bodies and dehydrated using 2-propanol (Thermo Fisher Scientific), cleared with methyl salicylate (Sigma-Aldrich), infiltrated and embedded in methyl methacrylate, and polymerized over 3–4 d. Sections of 4–6 µm thickness were cut using a sledge microtome (Leica SM2500, Leica Biosystems, Buffalo Grove, IL, USA) and mounted on charged slides. Sections were deplasticized by two rounds of incubation in xylene for 30 min followed by a 1 : 1 mixture of Xylene and ethylene glycol monoethyl ether (EGME, Thermo Fisher Scientific) for 5 min. After washing the sections with fresh EGME followed by tap water rinsing, the sections were stained with toluidine blue (Sigma-Aldrich) for 15 min, rinsed with tap water, and cover slips were placed on stained sections with mounting medium (Laudier *et al.*, 2006).

#### Statistical analysis

Data are presented as the mean ± SD. Repeated measures ANOVA with a Tukey’s *post-hoc* test were performed to compare biomechanical properties between all paired combinations (*i.e*., Intact *vs*. Injured, Intact *vs*. Hydrogel Implant/No Implant, Injured *vs.* Hydrogel Implant/No Implant) (*p* < 0.05 considered significant).

### Part III: Biological response

#### Cytocompatibility

Human dermal fibroblasts were seeded in CMC_90_MC hydrogels, polymerized with 20 mM APS/TEMED at a density of 12×10^6^ cells/mL and cultured for 6 d. Dermal fibroblasts were used as a general screen for connective tissue cytocompatibility, given the potential for CMC-MC gels to be employed for other clinical indications (*i.e*., soft tissue reconstruction), as well as for comparison to our prior studies evaluating related cellulosic gels (Gold *et al.*, 2014; Gold *et al.*, 2015). Direct-contact cytotoxicity testing by cell encapsulation was utilized since our previous experiments reveal poor cell adhesion and migration of NP cells, marrow-derived mesenchymal stromal cells and dermal fibroblasts seeded on CMC (Gupta *et al.*, 2011; Reza and Nicoll, 2010). This is likely attributed to the highly negatively-charged, hydrophilic environment of the CMC polymer, which results in poor serum protein adsorption due to strong water molecule binding to the material (Vogler, 2012). Similar models are employed by other research groups to assess cytocompatibility of biomaterials (Mironi-Harpaz *et al.*, 2012; Shin *et al.*, 2012). CMC (3 % w/v) hydrogels, fabricated using a 10 mM redox initiator concentration at the same cell-seeding density were used as controls, based on our earlier studies which show good cytocompatibility using this formulation (Varma, 2016). All hydrogels (*n* = 5) were cultured at 37 °C and 5 % CO_2_ in high glucose DMEM with 1 % Pen/Strep (Gibco, United States) and 10 % FBS (Gibco, United States). Total DNA content was measured to assess cell proliferation using the PicoGreen assay (Molecular Probes, Eugene, OR) on d 1 and 6. The samples were lyophilized, homogenized and digested in pepsin, prepared in 0.05 N acetic acid for 48 h at 4 °C (Sigma-Aldrich). The digested solution was then neutralized with 10× Tris-buffered saline. The samples were read on a BioTek Instruments plate reader (Synergy 4, Winooski, VT, USA) at an excitation/emission of 480/520 nm. Calf thymus DNA (Sigma-Aldrich) was used to create a standard curve (Gupta *et al.*, 2011). Additionally, the cell viability in the hydrogels was assessed on days 1 and 6 using Live/Dead assay (Invitrogen, Thermo Fisher Scientific, USA) staining with calcein AM and ethidium homodimer-1.

#### Biocompatibility

The foreign body response to the CMC_90_MC hydrogels, created with varying concentrations of APS and TEMED (0, 10 and 20 mM), was assessed using a subcutaneous pouch model in male Sprague Dawley rats (*n* = 4) (Charles River, Kingston, NY, USA) weighing 250–300 g. This resulted in the following groups: CMC_90_MC-0 (0 mM), CMC_90_MC-10 (10 mM) and CMC_90_MC-20 (20 mM) (Table 2). The CMC_90_MC-0 hydrogels lacking redox initiators served as negative controls, and gelled *in situ* purely by thermogelation and were not covalently crosslinked. Subcutaneous injection in Sprague Dawley rats is routinely used to characterize the foreign body reaction to polymeric biomaterials, and the size of the animal allows for clinically-relevant injection volumes (500–1000 µL) to be tested (Ibim *et al.*, 1998; Wallace *et al.*, 1992), as a prelude to evaluation in a large animal disc injury model. A 500 µL volume of the sterilized hydrogel solution was injected subcutaneously with a 20 G needle into each rat, at four sites on the dorsum, while under isoflurane-O_2_ general anesthesia, in compliance with a protocol approved by the Institutional Animal Care & Use Committee of The City College of New York. The rats were transferred back to their cages 30 min after injection to ensure adequate gelation of the polymer solution. They were fed a normal diet and monitored for changes in behavior and infection at the injection sites. At day 30, the animals were euthanized by CO_2_ asphyxiation, after which, the hydrogels were excised with the surrounding fibrous capsule intact and the foreign body response was assessed using histological methods. The study was conducted in accordance with ISO standard 10993-6:2007, to test for local effects of medical devices after implantation.

The isolated hydrogel samples were fixed in zinc buffered formalin and processed in EGME, followed by a second dehydration step using 2-propanol for 5 h. The specimens were cleared using methyl salicylate for 10 h and infiltrated with paraffin for 6 h, after which the samples were embedded in paraffin. Sections, 5–8 µm in thickness, were prepared using a Microm Rotary Microtome (Model HC 325; Thermo Scientific, Walldorf, Germany). Sections were deparaffinized using petroleum ether followed by EGME rinses. After hydration, the sections were stained with hematoxylin and eosin (H&E) (Sigma-Aldrich), alcian blue (Sigma-Aldrich) and picrosirius red (Polysciences, Inc., Warrington, PA). A polarized filter was used to view the picrosirius-red-stained samples to identify the birefringent collagenous capsules. For CD68 staining, deparaffinized and hydrated sample sections underwent heat-induced antigen retrieval in citrate buffer, and were stained with a mouse anti-CD68 antibody (Abcam, Cambridge, MA) (1:400 dilution) with a DAB-based chromogen to detect macrophages. In addition to non-immune IgG (Sigma-Aldrich) controls, rat spleen sections were used as positive controls. The images were captured using a Zeiss Axio Imager Z1 (Carl Zeiss, USA) optical microscope. The fibrous capsule thickness was measured using ImageJ (NIH) (Schneider *et al*., 2012).

## Results

Spectral analysis of modified CMC and MC polymers using ^1^H-NMR revealed a methacrylation modification of 15 % and 8 %, respectively (Fig. 1**a**). Preliminary assessment of MC polymer solutions at varying macromer concentrations demonstrated that a minimum of 3 % (w/v) was required to create a thermogelling network of MC at 37 °C. Therefore, all formulations of CMC were combined with a 3 % (w/v) MC hydrogel solution. Gross observation of the samples 30 min post injection suggested that a minimum of 20 mM redox initiators APS and TEMED, and a minimum of 3 % (w/v) CMC are required to provide consistent gelation and stable hydrogel formation within bovine motion segments. Additional evaluation showed that the increased viscosity of the methacrylated CMC-MC DPN compared to methacrylated CMC alone allowed for local retention of the injected material and subsequent curing *in situ* (Fig. 1**b**,**c**). Thus, two formulations at 6 % (w/v) of total polymer, CMC_90_MC (CMC (90 kDa, 3 % (w/v)) + MC (3 % (w/v)) and CMC_250_MC (CMC (250 kDa, 3 % (w/v)) + MC (3 % (w/v)), were chosen for material characterization using redox initiators at concentrations of 20 mM.

### Material characterization of *in situ* crosslinked hydrogels

**Fig. 3:**
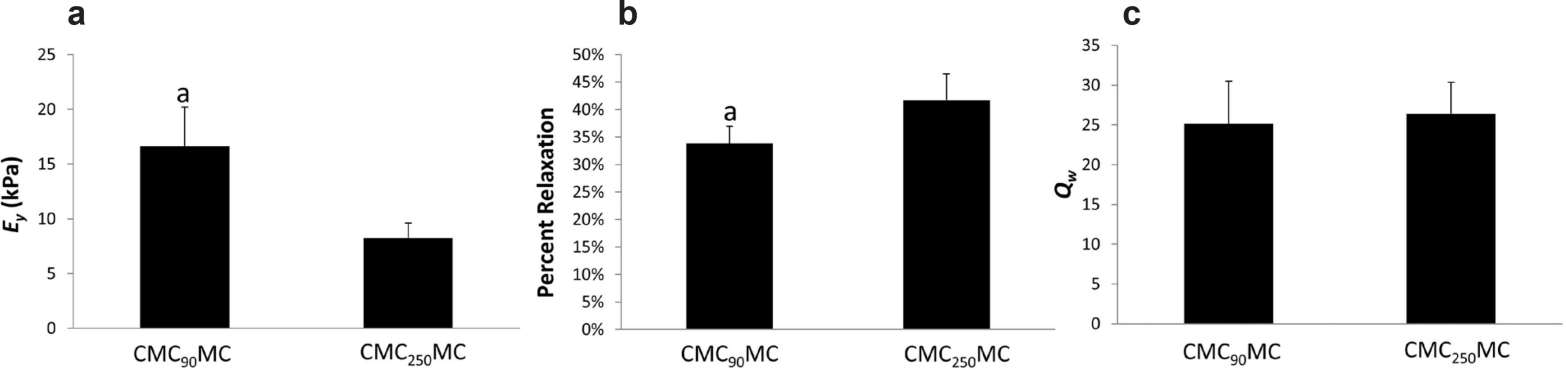
Material properties of CMC_90_MC and CMC_250_MC hydrogels created *in situ* within bovine motion segments. (**a**) Equilibrium Young’s Modulus (*E_y_*). (**b**) Percent relaxation (**c**) Equilibrium swelling ratio (*Q_w_*). ^a^Significantly different w.r.t. CMC_250_MC hydrogels.

Material properties of CMC-MC hydrogels formed *in situ* within bovine motion segments were evaluated. Initial rheological assessment of the methacrylated CMC_90_ and CMC_250_ base polymers highlighted the differences between their viscosities. The complex viscosity at 37 °C of CMC_90_ (0.02 ± 0.01 Pa × s) was significantly lower than CMC_250_ (0.32 ± 0.06 Pa × s). Nevertheless, the compressive mechanical properties of the crosslinked CMC_90_MC hydrogels following *in situ* gelation were better than those of the CMC_250_MC hydrogels, with a significantly higher equilibrium Young’s modulus (*E_y_*) of 16.62 ± 3.59 kPa and a significantly lower % relaxation of 33.82 ± 3.11 compared to the CMC_250_MC gels (8.249 ± 1.372 kPa and 41.708 ± 0.048, respectively) (Fig. 3). Furthermore, the *E_y_* (34.27 ± 4.53 kPa) of CMC_90_MC hydrogels formed *in vitro* in a custom casting device, was significantly higher than that of hydrogels formed *in situ* within motion segments. No significant differences were noted between the *Q_w_* of hydrogels from CMC_90_MC and CMC_250_MC formulations. Both formulations had an average *Q_w_* of ≈ 25 (Fig. 3**c**).

**Fig. 4:**
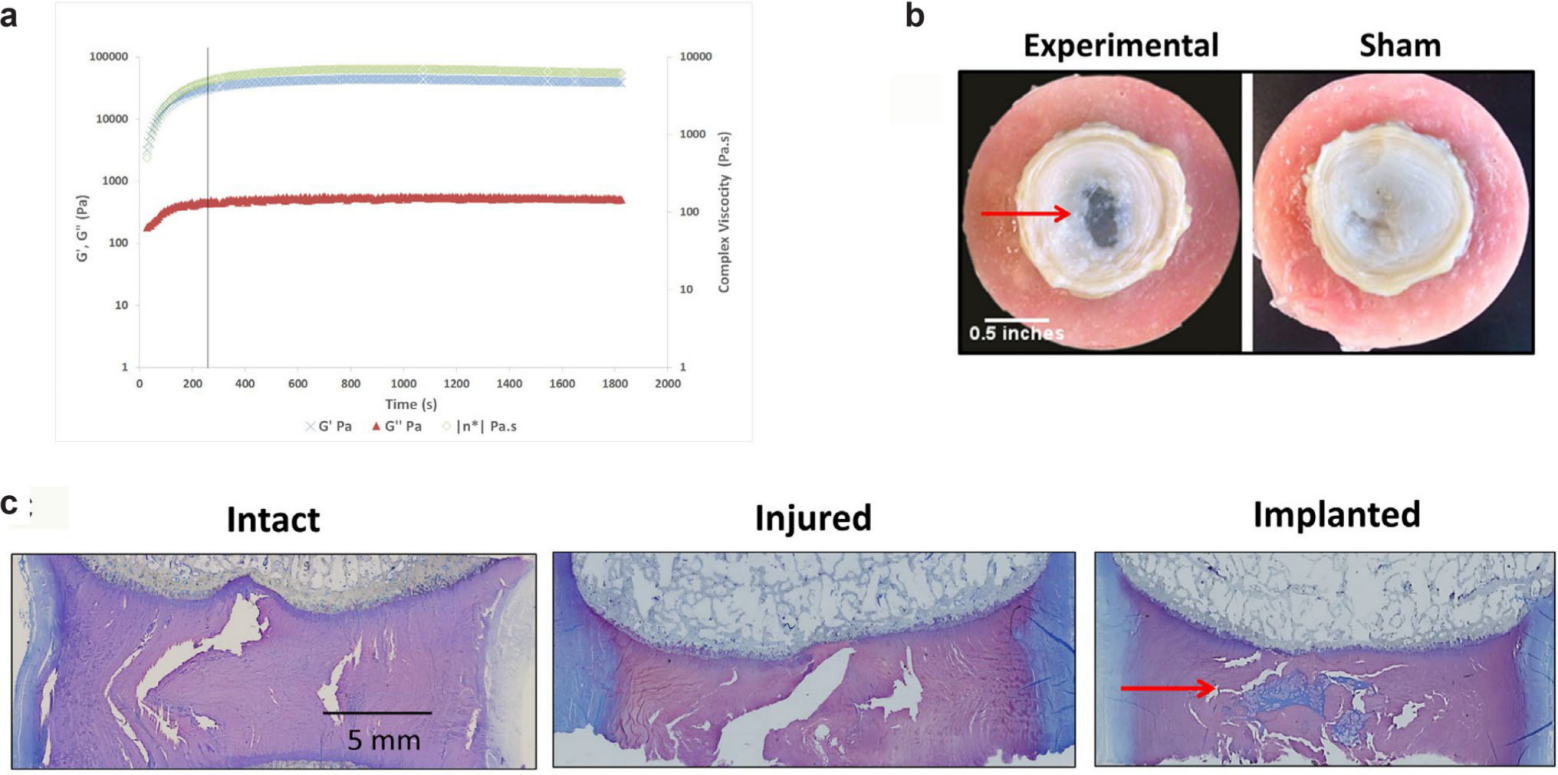
Gelation and post-injection characteristics of the CMC_90_MC hydrogel. (**a**) Gelation kinetics of the CMC_90_MC hydrogel represented by G’, G” and complex viscosity with time. Black line represents the point of gelation completion. (**b**) Gross images illustrating the presence of the CMC_90_MC hydrogel completely filling the NP void in the Experimental group contrasted with the void NP in the Sham group. (**c**) Histological sections of motion segments in the Intact, Injured and Implanted conditions stained with toluidine blue. Red arrow indicates the CMC_90_MC hydrogel filling the NP void space.

Based on the lower complex viscosity and *E_y_*, the CMC_90_MC formulation was further characterized. Rheometry at 37 °C revealed a gelation time of 3.96 ± 0.21 min (Fig. 4**a**) and a complex viscosity of 440.7 ± 166.4 Pa × s within 30 s upon loading the hydrogel on the instrument.

### Motion segment biomechanics

Biomechanical behavior of the motion segments was evaluated using a repeated measures design, where samples underwent testing under three conditions: Intact, Injured (post discectomy) and Implanted (with CMC_90_MC hydrogel). Gross observations of the bovine motion segments, upon completion of the mechanical testing regimen, revealed that the CMC_90_MC hydrogel implant filled the void NP region of the IVD (Fig. 4**b**). Changes in the IVD structure were visualized with toluidine blue staining of histological sections of motion segments under the three conditions: Intact, Injured and Implanted (Fig. 4**c**). The Injured samples exhibited a distinct void space as a result of the lost NP, while in the Implanted samples of the Experimental group, the CMC_90_MC hydrogel filled this void and interdigitated with the surrounding tissue.

### Effect of discectomy

**Fig. 5:**
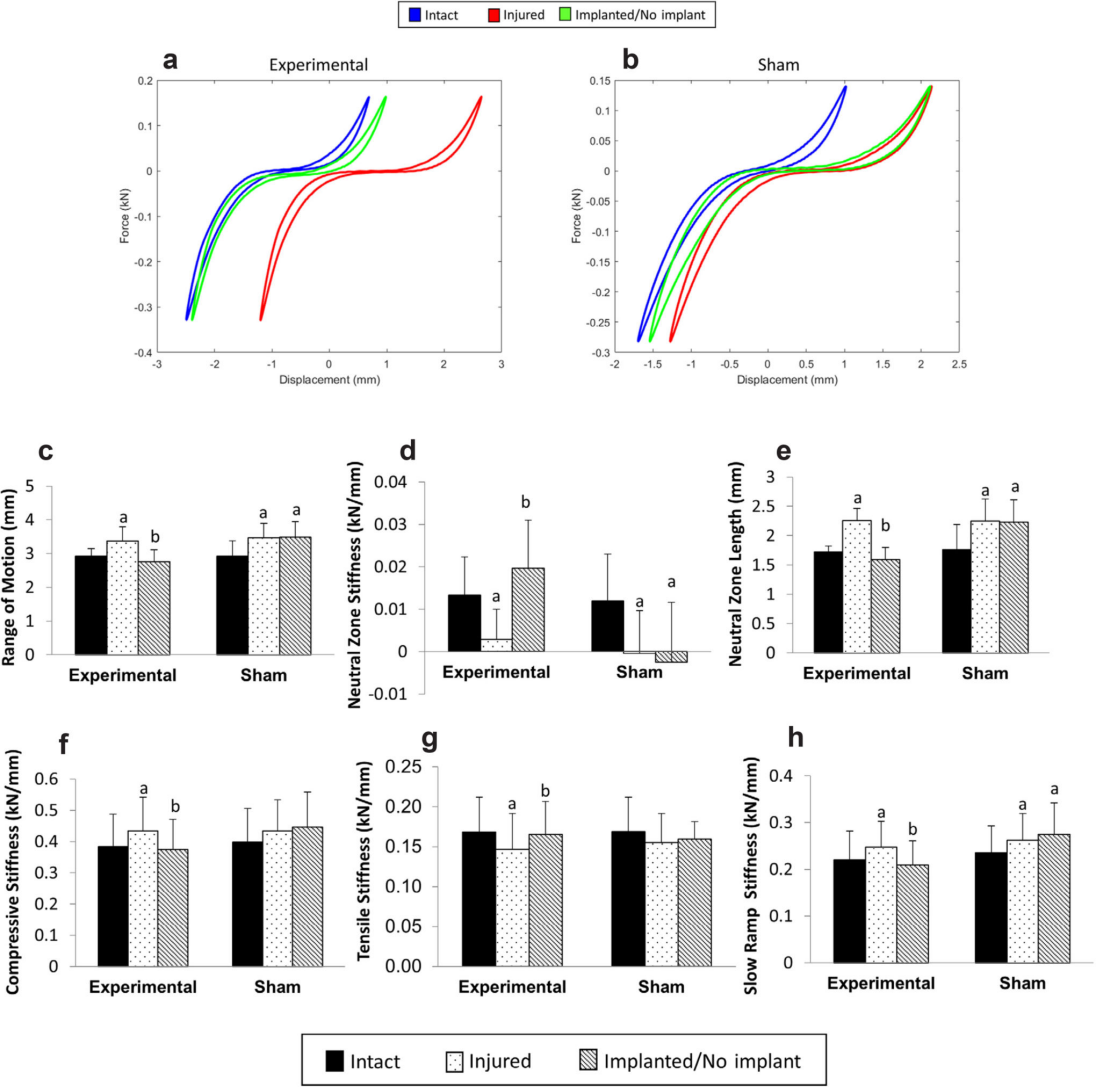
Biomechanical evaluation of motion segments with and without hydrogel repair. (**a**,**b**) Representative force-displacement curves of motion segment samples over the three-day test from the Experimental and Sham groups. Comparison of biomechanical parameters between Experimental and Sham groups in the Intact, Injured and Implanted/No Implant conditions (**c**) Range of motion (**d**) Neutral zone stiffness (**e**) Neutral zone length (**f**) Compressive stiffness (**g**) Tensile stiffness (**h**) Slow ramp stiffness. ^a^Significantly different w.r.t intact, ^b^Significantly different w.r.t injured *p* < 0.05.

**Fig. 6:**
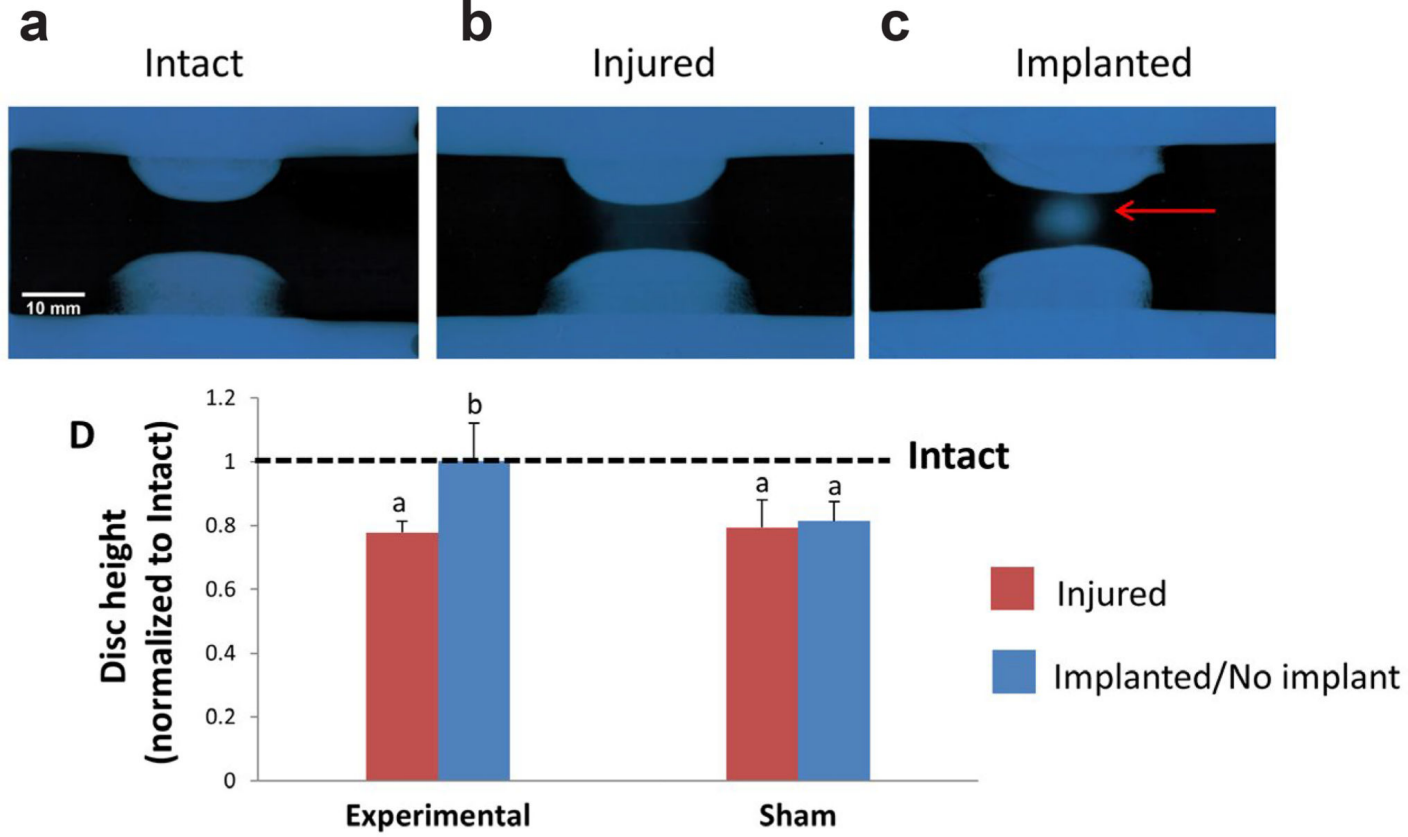
Changes in IVD height between Intact, Injured and Implanted conditions. (**a**,**b**,**c**). X-ray radiographs of explants at the three conditions. Red arrow in (**c**) indicates the presence of the CMC_90_MC hydrogel inside the implanted motion segment. (**d**) Numerical values of the IVD height were obtained from the radiographs, which were normalized to Intact IVD height values (dotted line). ^a^Significantly different w.r.t intact, ^b^Significantly different w.r.t injured *p* < 0.05

Injury by discectomy of motion segments, in both Experimental and Sham groups, resulted in significant changes in all measured biomechanical parameters, except for the compressive and tensile stiffness in the Sham group that did not exhibit a significant effect post injury (Fig. 5**f**–**g**). Representative force-displacement curves for both the Experimental and Sham groups are shown in Figs. 5**a** and 5**b**, respectively, displaying the effect of discectomy and hydrogel treatment on the curve profiles. In the Experimental group, the ROM of the injured samples increased by 15.5 %, from 2.92 ± 0.23 mm in intact to 3.37 ± 0.43 mm in injured specimens (Fig. 5**c**). Discectomy also resulted in a significant reduction in the NZ stiffness, from 0.013 ± 0.01 kN/mm in Intact to 0.003 ± 0.007 kN/mm in Injured samples (Fig. 5**d**). This was accompanied by a significant increase in the NZ length and slow ramp stiffness, from 1.73 ± 0.1 mm to 2.26 ± 0.21 mm (Fig. 5**e**) and 0.22 ± 0.06 kN/mm to 0.25 ± 0.06 kN/mm (Fig. 5**h**), respectively. Furthermore, injured motion segments suffered a ≈ 20 % drop in IVD height compared to Intact IVD height (7.68 ± 1.34 mm) (Fig. 6**d**), which can also be visually observed in the x-ray radiographs (Fig. 6**a**,**b**). No significant differences were observed in Intact and Injured conditions between the Experimental and Sham groups (Fig. 6).

### Effect of hydrogel treatment

The CMC_90_MC hydrogel did not extrude from the motion segments at any point during the axial mechanical testing or incubation. Upon injection, the implant significantly reduced the ROM in the Experimental group from 3.37 ± 0.43 mm in Injured to 2.76 ± 0.35 mm in Implanted specimens, thus restoring the ROM back to the Intact value (2.92 ± 0.23 mm) (Fig. 5**c**). The NZ stiffness of Implanted samples in the Experimental group was 0.02 ± 0.01 kN/mm, which significantly exceeded the Injured condition (0.003 ± 0.007 kN/mm), and recovered to Intact values (0.012 ± 0.009 kN/mm) (Fig. 5**d**). After hydrogel implantation, the compressive and slow-ramp stiffnesses significantly decreased compared to the Injured group, measuring 0.50 ± 0.13 kN/mm and 0.21 ± 0.05 kN/mm, respectively (Fig. 5**f**,**h**), while the tensile stiffness, 0.19 ± 0.04 kN/mm (Fig. 5**g**), was significantly higher than the Injured specimens. Additionally, the NZ length of the Experimental group was 1.59 ± 0.21 mm after hydrogel implantation, and was similar to Intact samples (1.71 ± 0.1 mm), while it maintained a high value of 2.23 ± 0.38 mm, similar to Injured (2.25 ± 0.38 mm) in the Sham group (Fig. 5**e**). Thus, all biomechanical parameters of the Implanted condition in the Experimental group were restored to the Intact condition. Conversely, most of the biomechanical properties in the ‘No Implant’ condition of the Sham group were significantly different from the Intact condition and similar to the Injured condition, with the exception of compressive and tensile stiffness, which did not show significant differences between the Intact and ‘No Implant’ conditions. Under the Implanted condition, restoration of the above biomechanical parameters was accompanied by recovery of IVD height to values in intact motion segments. The Experimental group exhibited no significant differences between Intact (7.76 ± 1.48 mm) and Implanted specimens (7.72 ± 0.87 mm), while the IVD height of ‘No Implant’ samples (6.19 ± 1.18 mm) of the Sham group was not significantly different from Injured motion segments (6.01 ± 1.11 mm) (Fig. 6**d**).

### Biological response

**Fig. 7:**
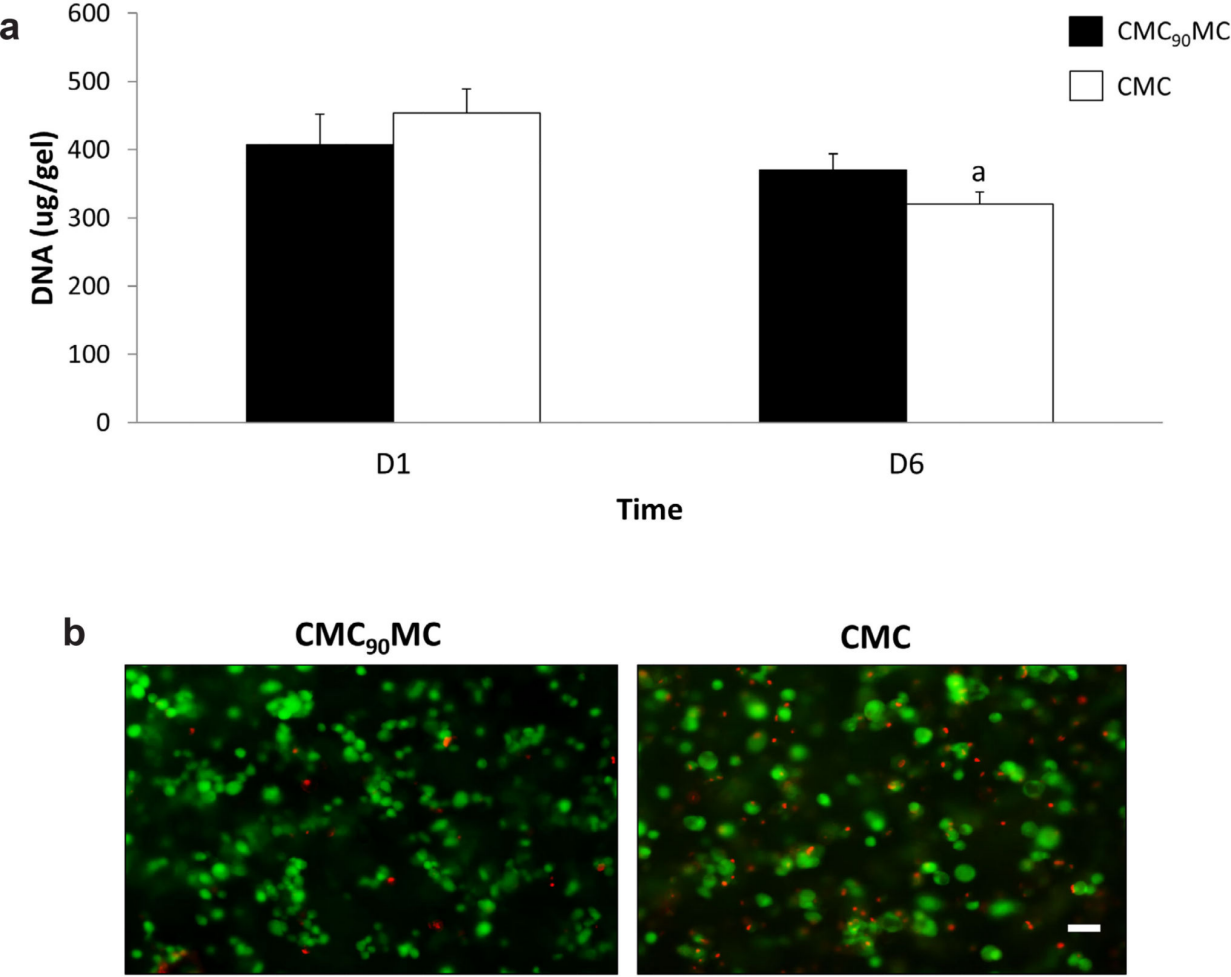
Cytotoxicity assessment of the CMC_90_MC hydrogel formulation. (**a**) DNA measurements quantified using PicoGreen on day 1 and day 6 in CMC_90_MC and CMC hydrogels. ^a^Significantly different w.r.t to day 1 (**b**) Live/Dead staining of the CMC_90_MC and CMC hydrogels on day 6, live cells stained in green and dead cells stained in red. Scale bar = 50 µm.

The cytocompatibility of the CMC_90_MC hydrogels used in this study, created with 20 mM of APS and TEMED, was tested *in vitro* on encapsulated cells over 6 d in culture. PicoGreen analysis of the DNA content in the gels showed no significant differences on day 1 and 6 between the CMC_90_MC hydrogels and the control CMC gels fabricated using 10 mM redox initiators, a formulation tested in previous cell-based studies (Varma, 2016). Further, qualitative assessment of cell viability by Live/Dead staining revealed that most of the cells were alive in both groups and were visually comparable through day 6 (Fig. 7).

**Fig. 8:**
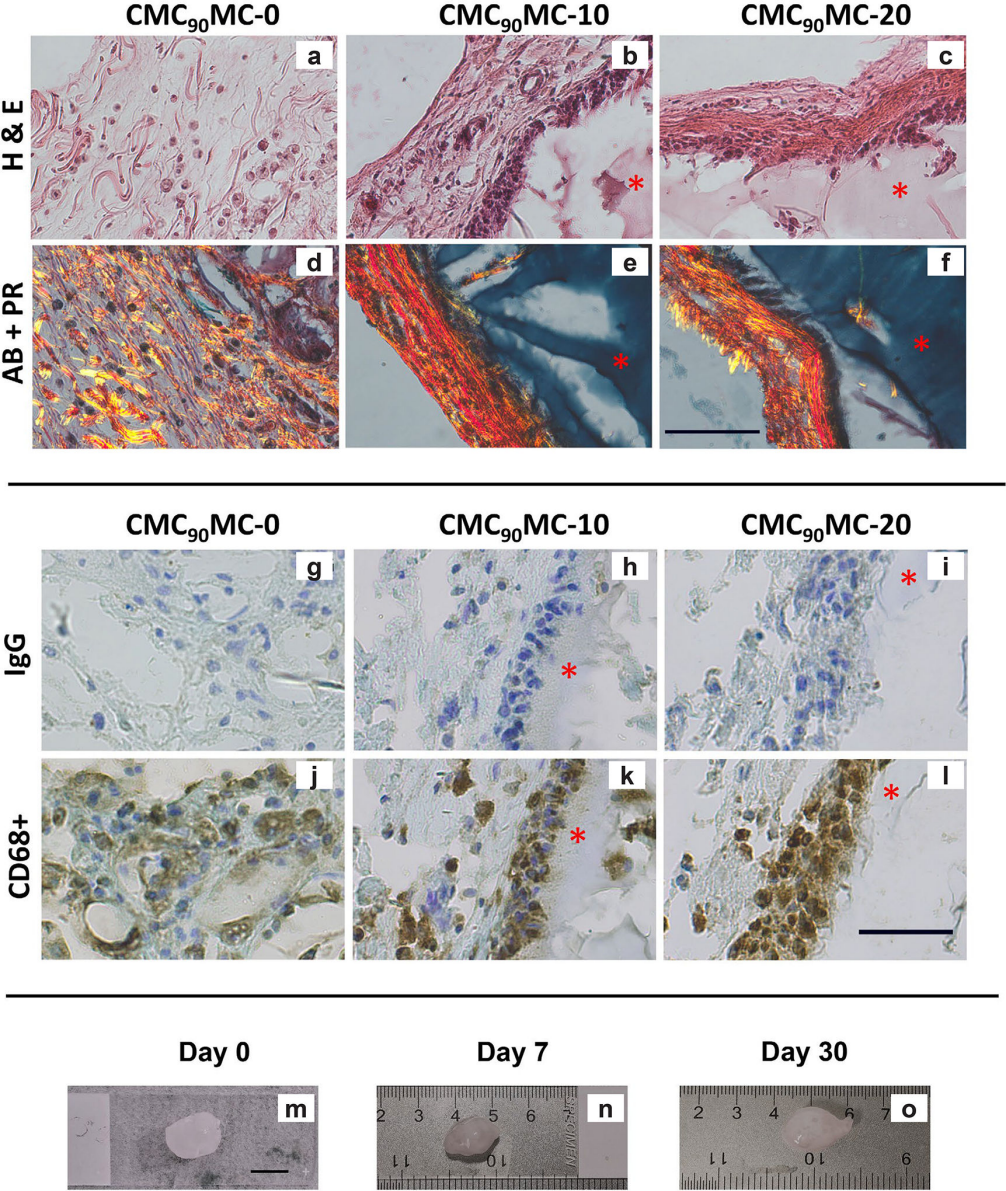
Top panel shows histological staining comparing the fibrous capsule thickness and composition in CMC_90_MC-0, CMC_90_MC-10 and CMC_90_MC-20 hydrogels isolated 30 d after subcutaneous injection *in vivo*. (**a**–**c**) H&E images depicting the fibrous capsule and the adjacent hydrogel. (**d**–**f**) Alcian blue (AB) stains the CMC_90_MC hydrogel while the picrosirius red (PR) highlights the collagen fibers in the fibrous capsule. Minimal blue staining indicates the remnants of the hydrogel in the CMC_90_MC-0 sample (**a**,**d**). The hydrogel is indicated by a red asterisk (*). Scale bar = 100 µm. Bottom panel displays immunohistochemistry staining of implants isolated at one month. (**g**), (**h**) and (**i**) are the IgG controls for CMC_90_MC-0, CMC_90_MC-10 and CMC_90_MC-20, respectively. Staining demonstrating the presence of CD68+ macrophages in (**j**) CMC_90_MC-0, (**k**) CMC_90_MC-10 and (**l**) CMC_90_MC-20 hydrogels. The hydrogel is stained blue with toluidine blue and is indicated by a red asterisk (*). Scale bar = 50 µm. (**m**–**o**) Gross images of CMC_90_MC-20 hydrogels isolated from the subcutaneous pouch of rats at different time points in the 30 d study. Scale bar = 10 mm.

The effect of redox initiator concentrations on the biocompatibility of the CMC-MC hydrogels was evaluated by comparing subcutaneously formed hydrogels with 0 (CMC_90_MC-0), 10 mM (CMC_90_MC-10) and 20 mM (CMC_90_MC-20) redox initiators. After 30 d of subcutaneous implantation *in vivo*, the rats had grown in size, displayed normal sleeping and eating habits, and exhibited no redness or inflammation at the injection sites. Overall, the animals appeared healthy and maintained normal mobility and behavior. The harvested CMC_90_MC-10 and CMC_90_MC-20 hydrogels were surrounded by fibrous capsules 111.29 ± 44.59 µm and 77.76 ± 28.45 µm thick, respectively. The CMC_90_MC-0 hydrogels did not reveal a distinct capsule and unlike the crosslinked gels, displayed cellularity and random arrangement of fibrous tissue within the hydrogel (Fig. 8**a**–**c**). These uncrosslinked samples were also too mechanically weak to handle and exhibited limited presence of CMC, as indicated by minimal alcian blue staining of the gels compared to the covalently crosslinked CMC_90_MC-10 and CMC_90_MC-20 hydrogels, which stained intensely blue and maintained their shape. Additionally, collagen fibers were highlighted by picrosirius red and were primarily limited to the fibrous capsule around the crosslinked hydrogels, while they were found randomly arranged throughout the CMC_90_MC-0 samples (Fig. 8**a**–**f**). Furthermore, CD68 staining of the CMC_90_MC-10 and CMC_90_MC-20 hydrogels exhibited a layer of macrophages adjacent to the hydrogel implants within the fibrous capsule, while the CMC_90_MC-0 hydrogels showed the presence of stained cells distributed within the diffuse material (Fig. 8**g**–**l**). The size and shape of the gels remained stable over the 30-day *in vivo* study based on the gross appearance of the implants (Fig. 8**m**–**o**).

## Discussion

There is a great need for NP replacement materials, since current treatments for degeneration with herniation commonly involve discectomy, which can cause loss of IVD height, alter IVD biomechanics and advance degeneration. This is the first study to report the development of a redox-initiated, thermogelling, crosslinked DPN of methacrylated CMC and MC. This novel DPN was injectable, gelled *in situ*, filled irregularly shaped voids, approximated native NP material properties, and restored the biomechanics of the IVD to healthy levels following discectomy.

### Polymer selection and hydrogel development

*In situ* gelation characterization of iterative formulations – prepared by varying the molecular weight of CMC, macromer concentration of methacrylated CMC and the redox initiator concentration – revealed two potential candidates as NP replacements: CMC_90_MC and CMC_250_MC. Since the CMC_250_MC hydrogels were composed of a higher molecular weight CMC, they were expected to have superior mechanical properties compared to the CMC_90_MC hydrogels. Surprisingly, the CMC_90_MC group had greater compressive modulus and lower percent relaxation following *in situ* gelation within the bovine IVD. The lower complex viscosity of CMC_90_ likely allowed for more uniform mixing of the CMC and MC polymers with the redox initiators; therefore, resulting in improved crosslinking and mechanical properties of the DPN. Thus, CMC_90_MC was chosen to be further tested for its potential to restore biomechanics of injured bovine motion segments.

The injectability of the CMC_90_MC solution may be ascertained by measurement of its complex viscosity (η*) (440.7 ± 166.4 Pa s), which was obtained within the first 30 s upon loading the hydrogel on the rheometer. This value is on the same order of magnitude as the η* of injectable dermal fillers available commercially, which range between 58–1199 Pa s (Falcone and Berg, 2008). Rheological analyses also determined the gelation time (3.96 ± 0.21 min) at 37 °C to be in accordance with the ISO standard 5833/1–1999 E for injectable materials (4–15 min), which is a distinct advantage over other *in situ* gelling systems that require several hours to achieve maximum mechanical properties (Smith *et al.*, 2014). The lower viscosity of the pre-gelled CMC_90_MC solution allowed penetration of the material into the cracks and fissures of the NP and AF, in addition to filling of the larger void spaces. However, histological analysis revealed incomplete filling of the smaller voids within the NP. While these smaller cavities could be artifacts from histological processing, this observation suggested that the infiltration of the hydrogel solution may be limited in regions of reduced connectivity, such as those likely associated with smaller voids. Future assessment may include microcomputed tomography measurements of the radiopaque hydrogel post injection to quantify filling of the NP deficit (Gullbrand *et al.*, 2017).

Comparing the mechanical properties of CMC_90_MC hydrogels formed *in vitro*, *versus in situ* revealed that the confined area of a casting device prevented loss of free radicals compared to the *in situ* environment of the NP void space, resulting in a significantly higher *E_y_* of gels formed *in vitro*. The *E_y_* values obtained by unconfined compression testing of both CMC_90_MC and CMC_250_MC gels formed *in situ* were higher than that reported for the native human NP tissue (*E_y_* of ≈ 5 kPa) (Cloyd *et al.*, 2007), although degeneration-related changes are expected to increase native tissue values (Umehara *et al.*, 1996). Furthermore, the CMC-MC hydrogel has an equilibrium compressive modulus similar to other NP replacement materials, which range between 9–35 kPa (Frith *et al.*, 2013; Smith *et al.*, 2014; Thomas *et al.*, 2010). Hydrogels of greater compressive strength might be necessary to withstand the loads experienced in injured or degenerated IVDs *in vivo*. Additionally, the CMC_90_MC hydrogels are expected to degrade hydrolytically *in vivo* due to hydrolysis of interchain ester crosslinks (Gupta and Nicoll, 2014; Reza and Nicoll, 2010; Varma *et al.*, 2014), potentially reducing their mechanical strength over time. As such, measuring the stability and degradation kinetics of these formulations *in vivo* would be an essential goal for future studies.

### Effect of discectomy and hydrogel implantation

Axial biomechanical behaviors of the motion segment were evaluated to detect the loss of NP pressurization and changes in the neutral zone (NZ), a parameter that describes the region of the IVD presenting minimal resistance to load; and, thereby, the portion of the force-deflection curve most sensitive to loss of pressurization due to NP degeneration or injury (Iatridis *et al.*, 2013; Panjabi, 1992). The effect of discectomy on bovine IVD motion segments was prominently demonstrated by the divergence of all biomechanical parameters in most of injured samples when compared to their intact values. The degree of injury in the motion segments was clinically relevant, as indicated by ≈ 20 % loss of IVD height post discectomy that is comparable to the 25 % disc height loss observed in human subjects undergoing discectomy (McGirt *et al.*, 2009). The ROM of all injured motion segments rose significantly from their intact values. As expected, the loss of the NP dramatically impacted the NZ, demonstrated by the significant reduction in NZ stiffness and increase in NZ length. Although the compressive and tensile stiffnesses of samples in the Experimental group changed significantly after discectomy, this effect was not consistent in the samples of the Sham group. This result could be attributed to the dependence of compressive and tensile stiffnesses predominantly on AF integrity (Johannessen *et al.*, 2006). The depressurization and AF destabilization during discectomy likely resulted in the significant reduction in tensile stiffness in the Experimental group, as reported previously (Likhitpanichkul *et al.*, 2014). Further, discectomy significantly increased compressive and slow ramp stiffness in the Experimental group, which suggested the transfer of load to the stiffer AF component of the IVD or to the cartilaginous endplates.

The IVD height is an important clinical parameter and an indicator of the level of hydration and hydrostatic pressure in the IVD. Thus, it was promising to observe that the injured IVDs regained their intact IVD heights after injection of the CMC_90_MC hydrogel. Moreover, all measured biomechanical parameters (ROM, NZ length, and slow ramp, compressive, tensile and NZ stiffnesses) were restored to intact values upon hydrogel injection, while the Sham samples showed no improvements from their injured values. Although transannular discectomy has been found to measurably alter the compressive and tensile moduli, these parameters are often not restored using an NP replacement alone (Balkovec *et al.*, 2013; Malhotra *et al.*, 2012; Smith *et al.*, 2014). Therefore, the recovery of these parameters with the CMC_90_MC hydrogel in the current study was unique but must be interpreted with some qualification since the Sham group displayed no statistically significant effect of discectomy on the compressive and tensile moduli.

Assessment of the NZ properties allows preferential measurement of the NP functionality in the IVD and is an important consideration for NP replacements. As expected, the NZ stiffness and length were restored back to Intact values with the hydrogel implant. Slow-ramp stiffness is proposed to reflect the mechanical response of the NP at low loads and low frequency (Johannessen *et al.*, 2006). In the current study, the removal of the NP reduced the viscous contribution to the low-load response region of the IVD and transferred the compressive loads to the surrounding AF and endplates, resulting in an increase of the slow ramp stiffness. Replacement of the lost NP with the CMC_90_MC hydrogel restored the hydrated, viscous state of the NP, lowering slow-ramp stiffness values.

This initial biomechanical study aimed to test the functional capacity of the CMC_90_MC hydrogel under axial compression and tension, yet there were some limitations that should be noted. The repeated measures design that was employed helped reduce the variability between samples originating from different bovine tails. However, this design also limited the direct comparison of hydrogel-implanted discs with their ‘No Implant’ counterparts. Past preclinical and clinical studies demonstrate the susceptibility of NP replacements to fail under physiological loading, especially in the lower back under bending and torsion (Panjabi and White, 1980). Thus, even though axial compression is a primary loading mode in the NP of the human IVD, additional evaluation of the hydrogel in other degrees of freedom, such as in bending, is essential – together with fatigue loading studies to assess resistance of the CMC_90_MC implant to herniation. Finally, although mechanical restoration of the NP is crucial to restrict progression of IVD degeneration, it is noteworthy that one of the primary failure modes of older generations of NP replacements has been by the annular injury post discectomy. Therefore, despite the fact that CMC-MC solutions can infiltrate into tissue cracks and fissures, including into AF tears surrounding the NP, parallel development of annular sealants or closure devices (Guterl *et al.*, 2013; Likhitpanichkul *et al.*, 2014; Parker *et al.*, 2016) may be important to retain this or other NP replacement biomaterials, should future rigorous biomechanical testing show evidence for herniation.

### Biological response to redox-polymerized cellulosic hydrogels

Independently, CMC and MC are established biocompatible polymers that are used in biomedical research and have an excellent safety profile with the US Food and Drug Administration (Miyamoto *et al.*, 1989). The biological response assessed in the current study aimed at monitoring the toxic effects of the redox initiators necessary to create stable, crosslinked hydrogels *in situ*. The *in vitro* cytotoxicity study revealed adequate cytocompatibility of CMC_90_MC hydrogels, as quantified by DNA measurements, in comparison to the control group consisting of previously developed cytocompatible CMC hydrogels prepared with a lower redox initiator concentration of 10 mM (Varma, 2016). The absence of a statistically significant increase in DNA content over time for both hydrogel formulations could be attributed to the limited proliferative capacity of anchorage-dependent fibroblasts in a hydrogel lacking binding sites (Guadagno and Assoian, 1991). The cytocompatibility results suggested the potential application of the DNP as a safe cell-delivery vehicle for biological repair of the NP, although additional evaluation of the material with more appropriate cell sources such as NP cells or mesenchymal stem cells would be necessary (Gullbrand *et al.*, 2017; Gupta and Nicoll, 2015).

Implantation into the rodent subcutis is a widely accepted model to test the biological response to new materials, as specified in ISO standard 10993-6:2007. In addition, the subcutaneous pouch model was selected over a disc injection model since it is difficult to yield conclusive results from small animal IVD testing; as the injection volume into the IVD is very small, technically challenging and may not represent the limited nutrition condition of the human IVD *in vivo* environment. Photocrosslinked MC hydrogels, tested in a murine subcutaneous pouch model, reveal a subtle foreign body response with a <80 µm capsule size (Stalling *et al.*, 2009). In the current study, the effect of redox initiator concentrations on the foreign body response to CMC_90_MC DPNs was studied by comparing the CMC_90_MC-20 hydrogels with CMC_90_MC-10 and CMC_90_MC-0 samples in rats. The fibrous capsules observed around the CMC_90_MC-10 and CMC_90_MC-20 gels indicated a modest biological response with a capsule thickness of 70–150 µm. This was comparable to responses seen with other biomaterials used for related biomedical applications (Bongio *et al.*, 2013; Deng *et al.*, 2010), and far less than the capsule thicknesses (>1000 m) associated with complications that require implant removal (Siggelkow *et al.*, 2003). Additionally, the presence of macrophages observed in hydrogels with (CMC_90_MC-10 and CMC_90_MC-20) and without redox initiators (CMC_90_MC-0) illustrated a normal foreign body response 30 d after implantation. With increasing implantation time, macrophages are typically found to recede, and the capsule becomes more defined, and exhibits aligned collagen and fibroblastic cells (Chiu *et al.*, 2009; Kim *et al.*, 2012).

Future biocompatibility testing in the subcutaneous space, with additional time points, will be necessary to observe the evolution of the initial biological response to redox-polymerized CMC_90_MC hydrogels. Qualitative assessment of the CMC_90_MC-20 hydrogels, retrieved from the subcutaneous pouch of rats, revealed negligible differences in the size of the hydrogels over several points in the 30-day study. This observation indicated minimal degradation of the hydrogels over the duration of the study. However, with time, the hydrogels are expected to degrade hydrolytically due to cleavage of the interchain ester crosslinks. Unlike other biopolymers, CMC-MC formulations will not undergo enzymatic degradation *in vivo*. Future work will include *in vitro* hydrolytic degradation studies of the CMC_90_MC hydrogels over a longer time, and subcutaneous implantation investigations using ultrasound to measure implant volume changes to quantify degradation *in vivo* non-invasively (Wortsman *et al.*, 2012). Finally, evaluation of the CMC-MC hydrogel in an *in vivo* large animal disc injury model will be necessary to examine its safety and biomechanical performance as an NP replacement material.

## Conclusions

The injectable, redox-polymerized CMC_90_MC hydrogel developed in this study combined the compressive mechanical properties and polyanionic character of CMC with the intrinsic thermogelling nature of MC to create a unique crosslinked DPN NP replacement biomaterial. The CMC-MC hydrogel exhibited strong potential as an NP replacement that could be injected in a minimally invasive manner to restore IVD height and compressive biomechanical function post discectomy. The biocompatibility studies also motivate additional assessments to advance towards clinical translation. Future studies are required to evaluate herniation risk under bending and fatigue loading and to eventually determine if IVD repair with this biomaterial can limit disease progression when implanted *in vivo*.

## Figures and Tables

**Table 1:** Formulations evaluated for *in situ* gelation. A range of polymer solutions was prepared by varying the molecular weight and concentration of macromers (CMC and MC), and the redox initiator (APS and TEMED) concentration.

Molecular weight (kDa)		Macromer concentration (w/v)		Redox initiator concentration (mM)
CMC	MC	CMC	MC	APS/TEMED
90	15:41	2%, 3%	3%	15, 20, 25
250	15:41	2%, 3%	3%	15, 20, 25

**Table 2:** CMC_90_MC formulations tested in the subcutaneous biocompatibility study.

Groups	APS and TEMED (mM)
CMC_90_MC-0	0
CMC_90_MC-10	10
CMC_90_MC-20	20
